# Exercise Capacity in Asymptomatic Adult Patients Treated for Coarctation of the Aorta

**DOI:** 10.1007/s00246-019-02173-5

**Published:** 2019-08-07

**Authors:** Elles J. Dijkema, Gertjan Tj. Sieswerda, Johannes M. P. J. Breur, Felix Haas, Martijn G. Slieker, Tim Takken

**Affiliations:** 1 Department of Pediatric Cardiology, Wilhelmina Children’s Hospital, University Medical Center Utrecht, Postbus 85090, 3508 AB Utrecht, The Netherlands; 2 Department of Cardiology, University Medical Center Utrecht, Utrecht, The Netherlands; 3 Cardiovascular Surgery, Wilhelmina Children’s Hospital, University Medical Center Utrecht, Utrecht, The Netherlands; 4 Department of Medical Physiology, Child Development & Exercise Center, Wilhelmina Children’s Hospital, University Medical Center Utrecht, Utrecht, The Netherlands

**Keywords:** Aortic coarctation, Exercise capacity, Hypertension

## Abstract

A reduced exercise capacity is a common finding in adult congenital heart disease and is associated with cardiovascular morbidity and mortality. However, data on exercise capacity in patients after repair of coarctation of the aorta (CoA) are scarce. Furthermore, a high rate of exercise-induced hypertension has been described in CoA patients. This study sought to assess exercise capacity and blood pressure response in asymptomatic patients long-term after CoA repair in relation to left ventricular and vascular function. Twenty-two CoA patients (age 30 ± 10.6 years) with successful surgical repair (*n* = 12) or balloon angioplasty (*n* = 10) between 3 months and 16 years of age with a follow-up of > 10 years underwent cardiopulmonary exercise testing at a mean follow-up of 23.9 years. Exercise capacity (peak oxygen uptake; VO_2peak_) and blood pressure response were compared to age- and gender-matched reference values. Left ventricular function and volumetric analysis was performed using cardiovascular magnetic resonance imaging. CoA patients showed preserved exercise capacity compared to the healthy reference group, with a VO_2peak_ of 41.7 ± 12.0 ml/kg/min versus 44.9 ± 6.7 ml/kg/min. VO_2peak_/kg showed a significant association with age (*p* < 0.001) and male gender (*p *≤ 0.001). Exercise-induced hypertension occurred in 82% of CoA patients, and was strongly related to left ventricular mass (*p* = 0.04). Of the 41% of patients who were normotensive at rest, 78% showed exercise-induced hypertension. No significant correlation was found between peak exercise blood pressure and age, BMI, age at time of repair, LVEF, or LV dimensions. Exercise capacity is well preserved in patients long-term after successful repair of coarctation of the aorta. Nevertheless, a high number of patients develop exercise hypertension, which is strongly related to systemic hypertension. Regular follow-up, including cardiopulmonary exercise testing, and aggressive treatment of hypertension after CoA repair is strongly advised.

## Introduction

Coarctation of the aorta (CoA) is a common congenital aortic lesion, which accounts for 5–10% of all congenital heart defects [1]. Untreated CoA may lead to morbidity and death early in life as a result of hypertension, congestive cardiac failure, myocardial infarction, stroke, infective endocarditis and aortic rupture [1]. Furthermore, despite timely repair, patients with CoA require life-long follow-up due to an increased risk of cardiovascular morbidity and mortality [1–3].This is largely due to CoA-associated hypertension which may be present in up to 68% at long-term follow-up [1, 3].

Reduced exercise capacity is a common finding in adults with congenital heart disease (CHD) and has been related to cardiovascular morbidity and mortality in this population [2, 3]. Subjective appraisal of exercise performance by CoA patients themselves is usually good. However, self-reported physical fitness is notoriously unreliable and frequently not in line with the actual physical fitness of the patient. Objective data on exercise capacity in patients after repair of coarctation are scarce [3]. A large number of CoA patients are normotensive at rest, but might develop hypertension during exercise [1, 4, 5]. Several studies have also shown a relationship between exercise-induced hypertension and systemic hypertension in CoA patients, suggesting that exercise-induced hypertension may be a predictor for the development of chronic hypertension and cardiovascular events in long-term follow-up [2, 4, 6, 7]. Hypertension has serious consequences for the future cardiovascular health since it may lead to atherosclerosis, coronary artery disease, stroke, left ventricular (LV) dysfunction, heart failure, and death [1, 3, 6]. Timely identification of patients at risk for hypertension is of great importance to initiate early intervention strategies and reduce the risk of hypertension related cardiovascular sequelae [1, 2, 4]. This study sought to assess exercise capacity and blood pressure response in adult patients long-term after CoA repair in relation to left ventricular and vascular function.

## Methods

### Study Population

All patients who received primary treatment (surgery or balloon angioplasty without stent placement) for localized CoA between 3 months and 16 years of age in our center between 1969 and 2004, with a follow-up of at least 10 years were invited to participate. Exclusion criteria were: isthmus hypoplasia (isthmus diameter < 40% of the diameter of the ascending aorta), aortic arch hypoplasia (proximal or distal transverse arch diameter < 60% or < 50% of the diameter of the ascending aorta, respectively) [8], or severe associated congenital heart defects (e.g., hypoplastic left heart syndrome, transposition of the great arteries). Of the 72 patients, 22 (31%) agreed to undergo cardiopulmonary exercise testing and cardio magnetic resonance imaging. The control group was based on age- and gender-matched reference values, obtained from the Low-lands Fitness Registry, a database containing CPET measurements of 2777 healthy Dutch people age 7 to 76 years [9] A control group of twenty-two controls was constructed by inclusion of a ‘reference control patient’ for each included CoA-patient, using the age- and gender-matched reference values for the analyzed CPET variables. This study was approved by the Medical Ethics Committee of the University Medical Center Utrecht (NL39345.041.12).

### Baseline Data

Baseline characteristics were obtained from the patients’ medical records and specifically designed questionnaires on medical history, current health, and cardiovascular risk factors, which patients filled out prior to the study date. Ambulatory blood pressure measurement was performed according to the European Society of Hypertension protocol [10]. Hypertension was defined as an systolic blood pressure SD score > 2 for children and a mean systolic blood pressure > 135/80 mmHg for adults [11, 12].

### Cardiopulmonary Exercise Testing (CPET)

CPET was performed on a calibrated, electronically braked upright cycle ergometer (Lode Corival, Lode bv, Groningen, The Netherlands), according to the Godfrey protocol, as cycle ergometry results in little noise in data collection [13]. Patients breathed trough a face-mask (Hans Rudolph Inc., USA) connected to a calibrated metabolic cart (ZAN 600, Accuramed bvba, Lummen, Belgium) during the entire test. A flow meter and gas analyzer for oxygen and carbon dioxide were used for volume measurements and breath-by-breath respiratory gas analyses. Oxygen uptake (VO_2_), carbon dioxide output (VCO_2_), and the respiratory exchange ratio (RER) were calculated automatically with conventional equations. Continuous measurement of the heart rate (HR) and oxygen saturation was performed with a 12-lead electrocardiogram (Spacelabs Cardioperfect, ItMedical, Veenendaal, The Netherlands) and pulse oximeter fitted on the forehead (Masimo Rad8, Masimo BV, Tilburg, The Netherlands), which was verified with the ECG heart rate. CPET data were only included when the test was performed to exhaustion with maximal effort, defined by subjective signs (i.e., sweating, unsteady biking) and objective signs (a peak RER > 1.0 or 1.1 for children and adults respectively). The test was terminated when the minimum required pedaling rate of 50 revolutions/min could not be maintained. Peak oxygen uptake (VO_2peak_) was calculated as the peak oxygen uptake averaged over the last 30 s of the test and corrected for body weight (VO_2peak_/kg; ml/kg/min). Maximal exercise data were expressed as a percentage of predicted (VO_2peak_/kg%pred) according to Dutch reference values that were derived with the same protocol and equipment. For peak blood pressure German reference values were used [14, 15]. Exercise-induced hypertension was defined as an age- and sex-specific systolic blood pressure above the 95th percentile, measured on the right arm [16]

### Additional Testing

CMR-imaging was performed using a 1.5 T scanner (Ingenia R4.1.2; Philips Healthcare, Best, The Netherlands). Left ventricular (LV) function and volumetric analysis, including LV ejection fraction (LVEF) and LV mass (indexed to body surface area), were performed using QMass Enterprise Solution (Medis Medical Imaging Systems, Leiden, The Netherlands). Body surface area (BSA) was calculated as described by Mosteller [17]

### Statistical Analysis

Statistical analyses were performed using IBM SPSS Statistics, version 25. Differences in outcome between CoA patients and controls were analyzed using a paired *T* test. An ANOVA was performed to compare hypertensive patients, normotensive patients and controls. The association between patient characteristics and VO_2_peak was analyzed using linear regression. Statistically significant variables with were included in a multiple regression model using forward stepwise selection. Test results with a *p* value less than 0.05 were considered statistically significant.

## Results

### Study Population

Twenty-two CoA patients were compared to age- and gender-matched reference values. Baseline characteristics of the study population are presented in Table 1. CoA patients were treated at a mean age of 5.9 years (range 0.35–14.4 years) with either BA (10 patients) or surgery with end-to-end anastomosis (12 patients). Mean follow-up at time of investigation was 23.9 years (range 10.8–42.2 years). At time of the investigation, a total of 13 patients (59%) were hypertensive, 8 (62%) surgical and 5 (38%) BA patients. Five (38%) hypertensive patients were receiving anti-hypertensive medication, 4 (80%) of them were still hypertensive. Nine (41%) CoA patients had a bicuspid aortic valve. Re-CoA had occurred in 5 (23%) CoA patients, 3 surgical and 2 BA patients. Re-CoA had been successfully treated with surgery, BA, or endovascular stent placement in 2, 1, and 2 patients, respectively. Median time between primary treatment and CoA re-intervention was 8.8 years (mean 13.8 years; range 2.1–40.4 years).

**Table 1:** Baseline characteristics of the study population

	CoA patients
Subjects	22
Age (years)	30 ± 10.6
BSA (m^2^)	1.9 ± 0.22
SBP (mmHg)	135 ± 13.2
DBP (mmHg)	76 ± 8.5
HR (bpm)	65 ± 11.0
Primary intervention	
Surgery	12 (55%)
BA	10 (45%)
Hypertension	13 (59%)
BAV	9 (41%)
Re-intervention	5 (23%)

*CoA* coarctation of the aorta, *BSA* body surface area, *DBP* diastolic blood pressure, *SBP* systolic blood pressure, *HR* heart rate, *BA* balloon angioplasty, *BAV* bicuspid aortic valve

### Cardiopulmonary Exercise Testing

Data on CPET are presented in Table 2. All patients reached a peak RER > 1.1, indicating maximal effort. Patients reached a mean peak workload of 3.45 ± 0.9 Watt/kg and a mean peak RER of 1.12 ± 0.08. No significant differences in peak oxygen uptake were found between patients and controls (*p* = 0.13). VO_2peak_/kg showed a significant negative association with age (*p* < 0.001) and a significant positive correlation with male gender (*p* ≤ 0.001) (Table 3). In multiple regression analysis, male gender and age both remained as a significant predictor of VO_2peak_/kg (*p* = 0.01 and *p* = 0.02, respectively). No significant correlations were found for age at time of repair, hypertension, exercise hypertension, or LVEF or LV dimensions. Patients showed a statistically significant higher peak blood pressure during exercise compared to controls (210 ± 21 mmHg vs 177 ± 3 mmHg, *p* < 0.01). Eighteen (82%) CoA patients showed exercise hypertension during CPET testing. Nine patients were normotensive at rest, of which 7 (78%) showed exercise-induced hypertension. Furthermore, patients showed a significantly lower peak heart rate compared to controls (180 ± 19 bpm vs 190 ± 5 bpm, *p* = 0.01). Two (9%) patients showed chronotropic incompetence, defined as failure to obtain ≥ 80% of the age-predicted peak heart rate [18].

**Table 2:** Cardiopulmonary exercise test results

	CoA	Control	*p* value
Age (years)	29.8 ± 10.6	30.0 ± 10.0	0.70
BMI (kg/m^2^)	24.5 ± 4.1	24.1 ± 2.0	0.64
Peak SBP (mmHg)	210 ± 20.9	177 ± 3.1	< 0.01
Peak HR (bpm)	180 ± 19.5	190 ± 5.4	0.01
Peak RER	1.12 ± 0.08	1.21 ± 0.04	< 0.01
VO_2peak_/kg (ml/kg/min)	41.7 ± 12.0	44.9 ± 5.7	0.13

*CoA* coarctation of the aorta, *BMI* body mass index, *SBP* systolic blood pressure, *HR* heart rate, *RER* respiratory exchange ratio, *VO2* max peak aerobic capacity, *Peak SBP* systolic blood pressure at peak exercise, *Peak HR* peak heart rate, *Peak RER* respiratory exchange ratio at peak exercise, *VO*_*2peak*_ peak oxygen uptake, *RPP* rate pressure product

**Table 3:** Regression analysis for VO_2peak_/kg

	Univariate analysis		Multivariate analysis	
	*B* coefficient	*P* value	*B* coefficient	*p* value
Male gender	15.79	< 0.001	10.03	0.01
Age (years)	− 0.56	< 0.001	− 0.33	0.02
BSA (m^2^)	− 13.27	0.26		
Age at repair (years)	− 0.70	0.21		
BAV	− 0.35	0.95		
EDV (ml/m^2^)	0.04	0.83		
ESV (ml/m^2^)	0.17	0.68		
LVmass (g/m^2^)	0.004	0.99		
LVEF (%)	0.14	0.80		
Hypertension	0.03	0.26		
Exercise-induced hypertension	− 4.46	0.12		
Re-intervention	1.59	0.80		

*BMI* body mass index, *BAV* bicuspid aortic valve, *EDV* end diastolic volume, *ESV* end systolic volume, *LVmass* left ventricular mass, *LVEF* left ventricular ejection fraction

Peak exercise blood pressure was significantly correlated to LV mass, 24 h ambulatory systolic blood pressure, and systemic hypertension (*p* < 0.05 for all). No significant correlation was found between peak exercise blood pressure and age, BMI, age at time of repair, LVEF, or LV dimensions. Hypertensive patients showed a significantly higher peak heart rate, compared to normotensive patients and controls. No significant differences in VO_2peak_ were observed between hypertensive patients, normotensive patients and controls. Patients who received BA showed a significantly higher peak heart rate, compared to surgically treated patients. No other differences in CPET measurements were found between patients treated surgically and patients who received BA. No differences in CPET measurements were found between patients with and without a bicuspid aortic valve.

## Discussion

Although self-reported exercise capacity in CoA patients tends to be good, objective data on exercise capacity after CoA repair are scarce [2, 3]. We found that exercise capacity is well preserved in adult CoA patients long-term after successful repair of coarctation of the aorta. However, exercise hypertension occurred in over two-thirds of CoA patients, and is strongly related to systemic hypertension. Despite a high rate of hypertension, cardiac function seems to be preserved during exercise.

Overall, CoA patients showed a good exercise capacity compared to controls. Peak oxygen uptake was similar between CoA patients and controls, as is in line with an earlier study by Balderston et al., who studied exercise capacity in 31 children after surgical repair of CoA [19]. Their cohort of patients with a mean age of 11 years showed a VO_2peak_ of 48 ± 1.4 ml/kg/min compared to a VO_2peak_ of 41 ± 12.0 ml/kg/min in our cohort with a mean age of 29.8 years, both with a VO_2peak_ predicted value of > 85% (89% vs 96%, respectively). In contrast, Buys et al. found impaired exercise capacity and lower VO_2peak_ in a large group of adults treated for CoA in childhood [2]. A recent study on CPET in children with CHD by Amedro et al. also showed decreased VO_2peak_ in a large cohort of CHD-patients with a mean age of 12 years [20]. A subanalysis showed a significantly lower VO_2peak_/kg for CoA patients compared to controls. However, CoA patients still reached 95% of the predicted VO_2peak_/kg [20].

Patients with CHD have been shown to experience a decline in exercise capacity from puberty, whereas VO_2peak_ continues to increase and VO_2peak_/kg remains stable in healthy adolescents during this period [21]. A retrospective cohort study by Diller et al. showed dramatically lower VO_2peak_y during cardiopulmonary exercise testing in patients with adult CHD [22]. In multivariate analysis, decrease in VO_2peak_/kg was identified as a strong predictor for hospitalization or death during follow-up. However, Diller et al. showed that the degree of exercise intolerance is related to the underlying anatomical features [22]. A smaller subanalysis showed that patients with repaired CoA demonstrated the highest VO_2peak_ values compared to patients with other complex CHD. Decrease in VO_2peak_ after anatomically successful repair was explained by associated lesions or late repair of CHD and therefore longer exposure to altered cardiovascular dynamics [3, 22]. The differences in reported exercise capacity might be explained by the heterogeneity in age, age at time of repair and associated lesions of the assessed study populations.

Increase of oxygen uptake during exercise is achieved through increased cardiac output (heart rate × stroke volume) and mixed arteriovenous oxygen difference, of which heart rate is the strongest contributor to the increased oxygen transport [18] Our patients showed a significantly lower peak heart rate than healthy controls and chronotropic incompetence was seen in 9% of CoA patients. Chronotropic incompetence has been strongly associated with increased risk of adverse cardiovascular outcomes and sudden death [18]. Although the underlying mechanisms for chronotropic incompetence in heart disease are not fully understood, reduced beta-receptor sensitivity or downregulation of beta-receptors as a result of chronic increased catecholamine levels have been implicated in patients with heart failure [18, 23]. Sino-atrial dysfunction has also been observed after cardiac surgery in patients with Tetralogy of Fallot [24]. Chronotropic incompetence after cardiac surgery might be explained by post-operative scarring of the myocardium. However, none of our patients with chronotropic incompetence had undergone open heart surgery, suggesting involvement of a more generalized arterio-ventricular pathophysiology.

Over two-thirds (68%) of CoA patients were hypertensive during exercise. This finding is in line with a recent study in children and adolescents after CoA repair [25]. Exercise hypertension is a strong predicator for systemic hypertension and cardiovascular events in long-term follow-up [2, 7]. Hypertension is a well-known long-term complication of CoA, even after surgical or endovascular repair. Reduced aortic compliance, abnormal baroreceptor function, and altered wall-shear stress dynamics have all been implicated in the development of CoA-associated hypertension [1, 2]. In a retrospective cohort study comparing CPET outcomes in normotensive and hypertensive CoA patients, Buys et al. showed that age was the only significant predictor for progression of exercise-induced hypertension to systemic hypertension [2]. Early detection and treatment of hypertension is essential in preventing long-term complications such as atherosclerosis, coronary and cerebral artery disease, heart failure, and death [26]. According to the recently updated guidelines of the European Society of Hypertension, patients with hypertension after CoA repair should be treated as particularly high risk patients, who require aggressive treatment with anti-hypertensive medication (beta-blockers or calcium channel blockers) and/or re-intervention in the case of rest- or restenosis [27]. As exercise-induced hypertension may precede systemic hypertension, regular follow-up of CoA patients, including CPET, is strongly recommended even long-term after successful repair [26, 27].

In regard to the results of this study, several limitations should be discussed. The relatively small sample size and low inclusion rate (31%) limits generalization of the results and may have obscured further differences and associations. Furthermore, the inclusion rate for the study was relatively low (31%), as a large number of CoA patients did not agree to undergo CPET in a study setting. This may have resulted in some degree of selection bias in the study. The control group consisted of age- and gender-matched reference values rather than actual control group participants. Nevertheless, these controls may provide a better comparison of CoA patients to a healthy standard than actual control participants, as exercise capacity can vary widely within a healthy population. Furthermore, as a large number of patients in our cohort were already hypertensive, comparison of patients with hypertension with patients with exercise hypertension only was not possible.

## Conclusion

Patients with coarctation of the aorta show preserved exercise capacity long-term after successful repair of the coarctation. Nevertheless, a high number of patients display exercise hypertension, which is strongly related to systemic hypertension and associated with adverse prognosis. Regular follow-up of CoA patients with cardiopulmonary exercise testing is recommended long-term after successful repair.
